# Do penguins care about their neighborhood? Population implications of bioerosion in Magellanic penguin, *Spheniscus magellanicus*, at Martillo Island, Beagle Channel, Argentina

**DOI:** 10.1371/journal.pone.0310052

**Published:** 2024-11-19

**Authors:** Gabriela Scioscia, Sabrina Harris, Adrián Schiavini, Klemens Pütz, Andrea Raya Rey

**Affiliations:** 1 Centro Austral de Investigaciones Científcas, Consejo Nacional de Investigaciones Científcas y Técnicas, Ushuaia, Argentina; 2 Wildlife Conservation Society, Buenos Aires, Argentina; 3 Antarctic Research Trust, Bremervörde, Germany; 4 Instituto de Ciencias Polares, Ambiente y Recursos Naturales (ICPA), Universidad de Tierra del Fuego (UNTDF), Ushuaia, Tierra del Fuego, Argentina; MARE – Marine and Environmental Sciences Centre, PORTUGAL

## Abstract

Intrinsic and extrinsic factors, such as bioerosion at nesting sites, regulate population dynamics and are relevant for the long-term conservation of penguins. Colony trends (between 2004–2022) were studied in a Magellanic penguin colony on Martillo Island, Beagle Channel, Argentina and compared between zones with contrasting degrees of erosion (high, medium, low). Individuals from each zone were characterized for foraging ecology, stress, and reproductive performance during the 2017–2018 breeding season to better understand the colony dynamics. Changes in nest abundance varied in magnitude between nesting zones with different characteristics of occupation time, density and erosion. Declines in nest abundance in the densest, most eroded and longest occupied zone suggests that environmental degradation may be limiting the colony’s carrying capacity. A higher percentage of late breeders (probably younger breeders) occupied the less eroded and more recently occupied zone. Foraging, breeding and stress barely differed between zones. New individuals recruiting into the breeding colony select less-eroded zones, either to reduce competition for nests or to avoid other effects of erosion and high-density areas. If this is the mechanism behind the shift in numbers throughout the island, we expect the island to be progressively occupied to the west. If competition or other density dependent factors are at play, a time will come when the vacant east side will begin to be recolonized by younger individuals. However, if erosion or other long-term effects spread throughout the island, recolonization may not occur and the colony may ultimately be abandoned as individuals search for new breeding grounds. Erosion at the breeding site may be a key factor in regional population trends of this burrow nesting species, by following an extinction / colonization of new sites process.

## Introduction

Seabird population fluctuations can reflect change in marine and coastal ecosystems [1, 2]. Knowing the factors that regulate populations are important for their conservation [3]. Population fluctuations are caused by complex interactions between intrinsic and extrinsic factors [4]. The way each factor contributes, and the intensity of these effects are species- and site-dependent [5–7]. Some extrinsic factors can affect seabird populations while individuals are in their breeding colonies. These include invasive or some native species, pathogens, human disturbance, climate change and nesting habitat [8]. Other extrinsic factors affect seabird populations while individuals are foraging at sea, for example bycatch, prey availability, changes in sea-surface temperature, pollution or overfishing [6, 8]. Intrinsic factors influencing populations include survival, productivity, ratio of breeders, density, recruitment and migration [9, 10].

Population fluctuations result from the interplay of survival and reproduction. In the case of long-lived organisms, the overall growth rate of a population is more influenced by adult survival than by factors directly related to their ability to produce offspring [11, 12]. Therefore, as adult survival is usually high in seabirds, population change tends to be slow [13, 14]. Philopatric species such as seabirds tend to breed in their natal colony [15, 16]. In addition, seabirds tend to breed in or close to the same nesting site year after year, therefore, recruits must occupy vacant nesting sites or fight to remove occupants of a desired nest [17, 18]. At a growing colony, new nesting sites are likely to be occupied by younger “naive” breeders [19].

Breeding performance over a breeding season is a relatively good estimator of individual success, yet other aspects such as variations in body condition throughout the season may provide a more complete picture of the reproduction costs and potential carry-over effects to future breeding attempts [20]. For long-lived birds under extreme environmental conditions or when resources are limited, adults must face trade-offs between their own survival and successful rearing of offspring [21–23].

Thus, fluctuations in the number of breeding pairs may be determined not only by adult survival, but also by the choice to breed in a given season, since breeders may skip some breeding seasons. The probability of breeding may be influenced by the past reproductive success [24], as well as the availability of resources such as food and nest site availability.

Nest type and quality are important factors affecting the reproductive success in penguins [15, 25]. The reduction of nesting sites is one of the primary threats to the conservation of many seabird species [26]. The loss of habitat available for breeding may result from elevated predation in certain breeding sites or from habitat modification due to human activities or introduced species [27–30]. In burrow-digging species, substrate type is important as burrows may collapse and flood in extreme weather events [31–34]. Seabird nesting behavior also modifies the breeding habitat through physical and chemical disturbances that alter edaphic conditions and can transform vegetation, especially in arid and sub-Antarctic sites [35–37]. This alteration is mainly produced through guano deposition, but also by the digging of burrows and/or trampling [36].

Breeding success in seabirds is also intimately linked to foraging success of breeders [38, 39]. Foraging success in turn is influenced by the age and experience of the breeders [40, 41]. Thus, in addition to the terrestrial factor, marine effects over the populations can be evaluated, considering the trophic habit of the animals nesting in areas with different conditions (*i*.*e*. erosion). Taken together, these characteristics, coupled with their breeding performance, will allow us to understand the population dynamics of a colony.

Magellanic penguins *(Spheniscus magellanicus)* breeding on Martillo Island (Beagle Channel, Argentina), are interesting subjects for population studies. They are long-lived philopatric seabirds that return to their breeding colony for successive breeding events, typically after reaching 3–4 years of age [15; Scioscia et al. data not pub.]. Another important trait is their nesting behavior, which commonly involves burrowing under bushes and has led to erosion of their breeding sites. In this colony of Magellanic penguins, caves used as burrow-nests, bridges, cracks, pedestals and trails were recognized as erosion features created by penguins [42]. In addition, muskrat (*Ondatra zibethicus*) inhabits the island and also create tunnels and feeds on vegetation, thereby further increasing the erosion [43]. This colony is monitored yearly since 2004 [44], therefore nest occupation and density has been estimated every season for each sector of the island and classified by its erosion level as identified in 2016 by [42]. Eroded sites may increase the risk of burrow collapse, flooding and even favor the prevalence of ectoparasites [45].

In this context, the objectives of this study were to: 1) assess the impact of the substrate erosion on changes in colony size of Magellanic penguin (*Spheniscus magellanicus*) on Martillo Island and, 2) characterize the individuals that inhabit each zone of the island with different degrees of erosion, in terms of their body condition, feeding behavior and preferences and reproductive performance, and 3) assess the implications of 1) and 2) on the demography of the colony and regional population.

## Methods

### Study area

**Fig 1:**
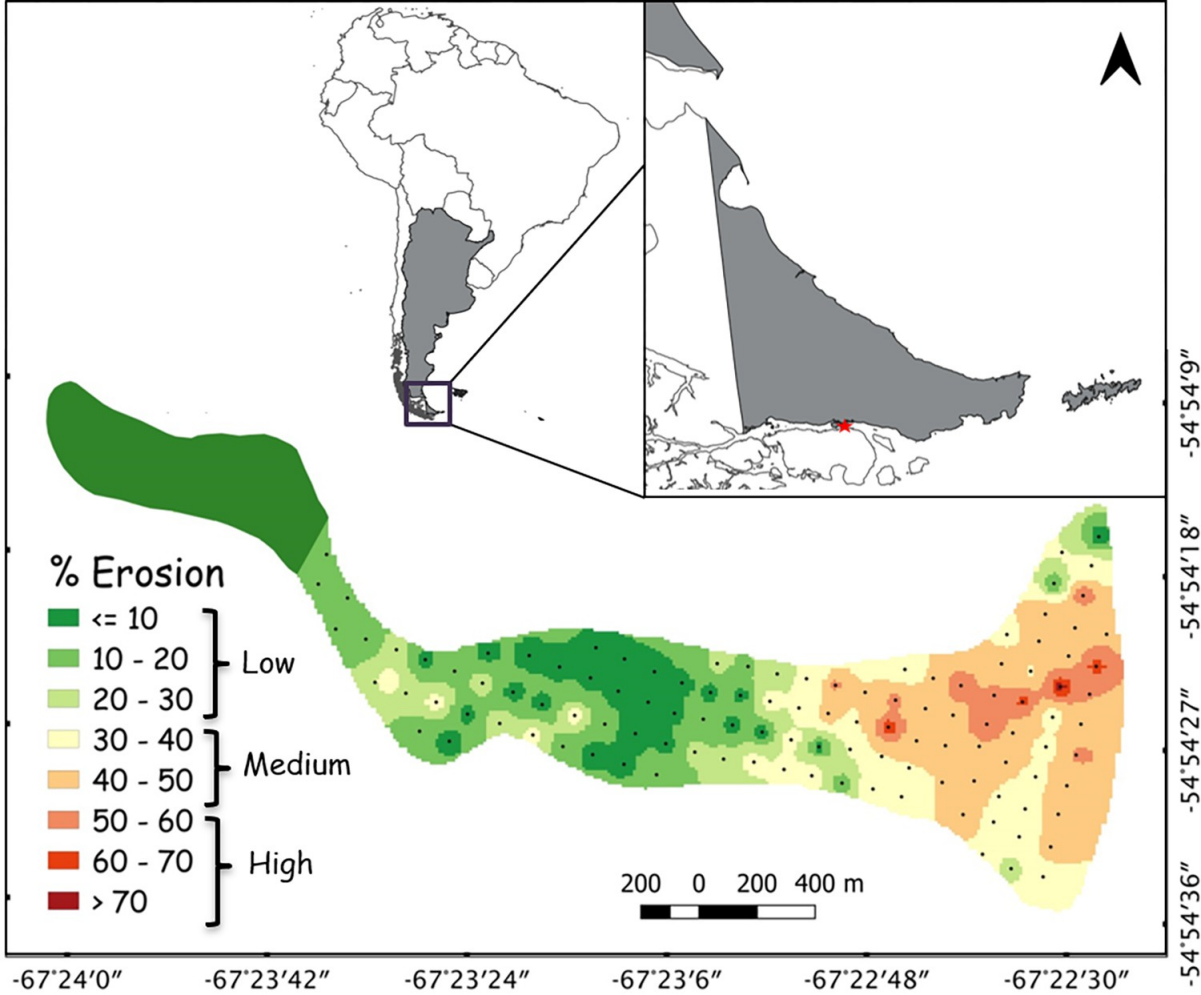
Bio-erosion categories at Martillo island, Beagle Channel, caused by Magellanic penguin (*Spheniscus magellanicus*). Zones with low (0–30%), medium (30–50%) and high (> 50%) erosion percentages of the island during 2016 year (modified of [42]). Dots: point of the permanent grid. Location of Martillo Island (red star in upper maps) in the Beagle Channel, Tierra del Fuego, Argentina (grey).

This study was conducted at the Magellanic penguin breeding colony on Martillo Island, Beagle Channel, Tierra del Fuego, Argentina (54° 54’ S, 67° 22’ W; Fig 1). Given the bioerosion of the island identified in [42], we re-classified it into three strata based on the degree of erosion: the low zone (0–30%), the medium zone (30–50%) and the high zone (> 50%) of degradation on the island (Fig 1 modified from [42]). The very high erosion area and the high erosion area (as indicated in [42]) were combined in this study, since the first was very underrepresented. Zones with a low degree of erosion were the most commonly represented on Martillo Island, occupying the central and western area and in some sectors towards the center of the south coast. The zone with medium degree of erosion occupied mainly the eastern sector, along cliffs, at the top of the eastern drumlin and in its northern slope and canyon. Lastly, the zone with higher degree of erosion occupied a narrow area in the center of the eastern sector [42].

The contour shapefile of South America, Argentina and Tierra del Fuego were obtained from the National Geographic Institute of the Argentine Republic (IGN), https://www.ign.gob.ar.

### Nest density, abundance estimates and colony size trend

#### Nest density and abundance estimates

A permanent point grid was established in 2004 (distance between points 50 m, Fig 1), and erosion degree (extracted from the stratum it belonged to), nest density and abundance were assigned to each point. Nest density (Dn: nests/ha) was estimated at each point using the point-transect method by counting the number of active nests within circular plots of 18 m radius during the egg laying period (end of October) for each breeding season from 2004 to 2022 (except 2007 and 2020, for logistical reasons). To produce density maps for the entire colony, the nest density per year and for each point was extrapolated to the entire colony area by means of the triangulation and interpolation of the values by point, using geoprocessing tools in QGIS 3.6 platform. The overall abundance estimate was calculated as the mean of the stratum (zone) estimates weighted by stratum area. In 2015 the grid was expanded to the western part of the island (Fig 1), following the expansion of the colony observed in 2014. The abundance estimation was carried out using Distance 7 Release 1 [46] with uniform detection function model. The confidence interval for the estimates was assessed empirically assuming a Poisson distribution of observations.

#### Colony growth trend and growth rate index

The colony growth trend from 2004 to 2022 was evaluated for each erosion zone. The data from the time series of the observed counts for each point each year were combined with the missing counts (*i*.*e*. year 2007 and 2020) and a log-linear regression model was fitted with Poisson error terms using the package *rtrim* (Trends and Indices for Monitoring Data TRIM, version 2.1.1; [47] in R software (version 4.2.2). TRIM estimates a piecewise loglinear growth model to compute imputations (either actual count or estimated if missing). We used a model that allows for changing trends over time, if populations vary across sites but exhibit the same growth pattern. Different growth rates are incorporated by introducing change-points in the equation, allowing for gradual shifts in population growth over time. The equation combines different slope parameters (β) based on the specified change-points, capturing the changing trends. For this, initially we applied a model with change-points (or knots: K), i.e. where the slope changed, at each time-point (all years) and used the autodelete selection to combine the time segments if there were not enough observations present in some time-point for a model to be estimated (because we had no survey data in 2007 and 2020 due to logistics issues). We considered over-dispersion and serial correlation, since they can affect standard errors, although they have usually a small effect on the estimates of parameters [48]. The estimation method used in *rtrim* is based on generalized estimating equations (GEE) [48]. The numbers of expected breeding pairs (*μ*_*ij*_) in each site *i* and year *j* were modeled as:

$$ln\mu_{ij}=\alpha_{i}+\sum_{l=1}^{L}(\beta_{l}-\beta_{l-1})(j-k_{l}){K(j,k}_{l})$$

where *α*_*i*_ is the effect for site *i*, with slope *β*. The time points or years when the slope parameter *(β)* changes are called change-points or knots and are denoted by *K*_*l*_, with *l* = 1 and *L* the number of change-points. Where *β*_0_ = 0 and the function *K (j*, *k)* is defined by *K (j*, *k)* = 0 for *j ≤ kl* and *K (j*, *k)* = 1 for *j >kl*. The growth rate (λ) is derived from λ = *e^β^* [48].

To assess if the habitat erosion had an impact on the trends, we used zone as a covariate in model 2. To identify change–points with significant changes in slope and the effect of covariate on the slope we used Wald tests with a significant-level threshold value of 0.05 [48].

#### Inactive nest index

To evaluate whether nest desertion was mediated by the degree of erosion we compared the average of the percentages of empty nests (± standard deviation) for each eroded zone (high, medium and low) and for the 2016, 2017, 2018, 2019, 2021 and 2022 breeding seasons. Normality and homoscedasticity of the data were assessed using the Shapiro-Wilk and Levene tests, respectively. Differences between erosion zones were examined using the Kruskal-Wallis test, followed by multiple comparisons to examine pairwise group differences using Dunn’s method (with Bonferroni correction).

### Body condition and health parameters

During 2017 breeding season, before the laying of the first egg (Pre-laying) and at the end of the late chick-rearing breeding stage (CR) adults were captured by carefully removing them from their burrows and weighed using a 10-kg Pesola balance (to the nearest 100 g). To estimate sex and body index of individuals, bill depth and length and flipper length were measured (followed [49, 50]. The body size index for breeding adults (b) was calculated as the first factor extracted from a principal component analysis on measurements of bill length, bill width and flipper length. This first factor explained 78% and 80% of the variance during the beginning (Pre-laying) and at end of the late chick-rearing stage (CR), respectively. The residuals for the mass x body size index regression (mass = 4.67 + b * 0.01 and mass = 4.10 + b * 0.24, for Pre laying and CR, respectively) were then used as indices of body condition. Differences in indices of body condition among zones and sexes were assessed using a two-way ANOVA for each breeding stage. Then, in order to compare the body condition throughout the breeding season, breeding stage was included as a fixed factor for each zone and was analyzed using ANOVA [51].

To complement the evaluation of body condition, the stress level of the adults was analysed by comparing the percentage of heterophiles vs. the percentage of lymphocytes (H/L) [52]. Blood samples were obtained at the beginning of the breeding season, before egg laying (Pre-laying), and during chick rearing (chicks between 30–50 days old, when adults were captured for device removal). Blood samples were made to blood smears *in situ* and later in the laboratory, smears were fixed in ethanol 96% and dyed with Giemsa stain (1:7) in distilled water for 15 min. Smears were examined under an optical microscope (Leica DM2500) at 1000x magnification with immersion oil, and leucocytes were classified and counted. The ratio of heterophiles/lymphocytes was estimated for each sample (H/L). A generalized linear mixed effect model (GLMM) was adjusted with the log10 transformed H/L ratio as the response variable, with stage (“Pre-laying” or “CR”), erosion level (“low”, “medium” and “high”) and their interaction as fixed effects and identity as a random effect (random = ID).

### Foraging behavior and trophic parameters

Trophic parameters were studied in the 2017 breeding season. During later chick rearing, 18 adults (10 female and 8 male) rearing chicks of between 30–50 days old (6, 7, and 5 from zones with high, medium and low degree of erosion, respectively) were captured at their nest and equipped with GPS loggers (GPS-TDlog, Earth and Ocean Technologies, Kiel, Germany) which recorded depth, latitude and longitude. The devices were attached to lower back feathers with Tesa tape (following [53]). After a period of between 2 and 3 days, once birds returned to their nest, the devices were recovered.

Devices recorded GPS fixes at 1s interval when individuals were at the surface and temperature and depth data every second throughout the trip, with an accuracy of 0.005°C, and 3.5 cm, respectively. Only one trip per individual was used in the analysis. Dive parameters were analyzed using MultiTrace (Jensen Software Systems, Kiel, Germany). A dive was deemed to have occurred if its maximum depth was >1 m. The rest of the criteria for the analysis of the trip were set following [50]. We calculated foraging trip duration (the sum of the durations of all dives and surface intervals between dives), dive rate (number of dives divided by foraging trip duration), percentage of time diving (sum of dive duration divided by foraging trip duration) and time spent at the bottom per foraging trip (bottom time: as the sum of bottom time duration for all dives divided by trip duration). In addition, we estimated maximum distance from the colony and path sinuosity (as the ratio of the total displacement, i.e. the sum of the distances of each track for the whole trip, to the linear displacement, i.e. two times the maximum distance to the colony) using geoprocessing tools in QGIS 3.10. These parameters were analysed using ANOVA test to evaluate for differences among zones. Both sexes were pooled in the analysis as no differences were found in any foraging trip parameter between males and females.

For stable isotope analysis, a blood sample was extracted from the tarsal vein and preserved in ethanol 70% for future carbon and nitrogen stable isotope determinations during the 2017 breeding season when adults were captured for device removal from zones with different degrees of erosion (high: n = 7, low: n = 6 and medium: n = 9). In addition, blood samples of 14 fledging chicks were obtained during late January in the zones with high (n = 9) and medium (n = 5) degree of erosion. Stable isotope values in blood reflected foraging ecology and diet within the three to four weeks prior to sampling [54]. Blood samples were preprocessed according to [55] and analyzed at Louisiana State University (USA). Stable isotope values were run with standard values for each isotope and are expressed in δ notation in per mil units (‰). The differences in δ^13^C and δ^15^N values among zones with different degree of erosion were evaluated with ANOVA test. Standardized ellipse areas corrected for small sample size (SEA_C_) were used to visualize the isotopic niche overlap based on δ^13^C and δ^15^N values were estimated using SIBER ([56, 57]; in R software version 1.0, R Core Team 2018). SEA_C_ was fitted to 40% of the data to represent isotopic niche width for adults and chick and for each erosion zone [57].

### Breeding parameters

At the beginning of the 2017 breeding season, during pair formation (late September to early October), 274 nests were marked and monitored at intervals of between two and six days, until egg laying was completed or until the nest was deserted, and the laying date and number of eggs were recorded.

We categorized each nesting pair as early, medial, or late breeders, based on whether they laid the first egg before or after the median date range of egg laying for the current season. During the 2017 breeding season the laying date peak occurred between October 10 and 15 (S1 Fig). The penguins that laid their eggs then were called medial breeders. The birds that laid their eggs before October 10 were named early breeders and those that laid after October 15 were named late breeders. The percentage of each class of breeders was assessed for each zone. The distribution of these variables and their association with the erosion zone was analyzed through a Chi-square test (X^2^).

Breeding success was assessed as the number of fledged chicks per nest (Ch/N). A chick was considered fledged according to their weight, molting status and age following [50]. The weight and wing length of the chicks was registered the last time that they were seen in the nest in advanced molt (late January to early February). The body index of fledging chicks was calculated as weight to wing length ratio.

Weight and body index of fledging chicks were compared among erosion zones using an ANOVA test. In addition to comparing breeding success in different zones of the island, we tested whether there was an effect of parental pre-laying body condition using a generalized linear model with Poisson distribution.

To verify if the data attend the assumptions of normality and homogeneity of variances, data were tested with Shapiro-Wilks and Levene’s test. All statistical analyses were performed using InfoStat software [56]. Means are presented with their standard deviations.

All applicable international, national, and/or institutional guidelines for the care and use of animals were followed. This study was evaluated and approved by the Wildlife Direction, Environmental Secretary, Tierra del Fuego Government taking into account animal research ethic perspective with Argentinian Government permission: Resol. SUB. P.A.y S N° 0063–17). “Trophic ecology of the seabird assemblage of the Beagle Channel and Isla de los Estados: spatial and temporal variation”.

## Results

### Nest density, abundance estimates and colony size trend

**Fig 2:**
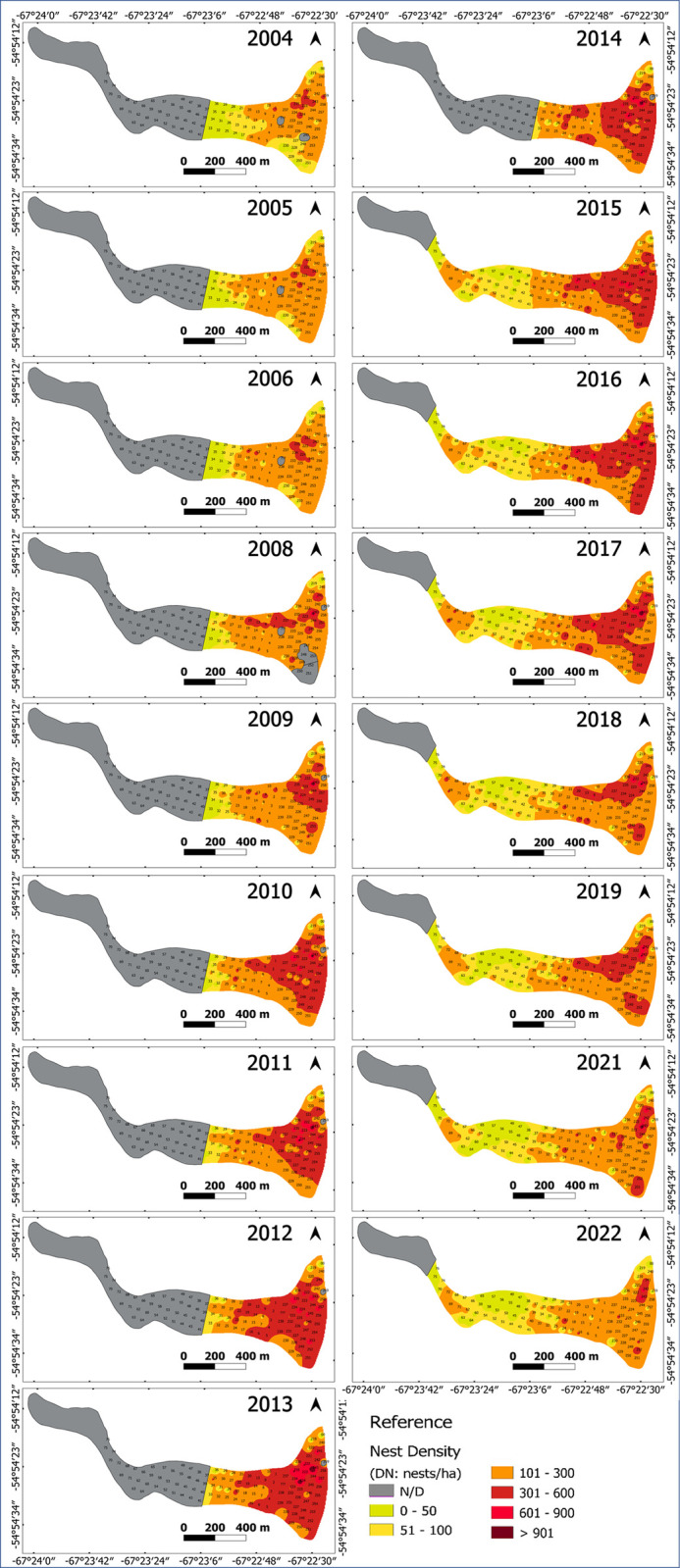
Nest density (Dn: nests/ha) of the Magellanic penguin colony in Martillo Island each year, from 2004 to 2022.

At the onset of the study period (2004–2006), the highest nest density corresponded to the northeast sector of the island, whereas in the west the density of nests was lower and there were no nests at all towards the western point. (Fig 2). Breeding was observed during 2014 in the western sector, where 375 nests were recorded by direct count in January 2015. Between 2008 and 2014, the highest density area gradually increased towards the southeast and west of the island, until 2014, when it retracted, but still being more extensive than before 2008. From 2017 onwards, the decrease in high-density areas was more pronounced, becoming smaller than in 2008 by 2021 (Fig 2).

**Fig 3:**
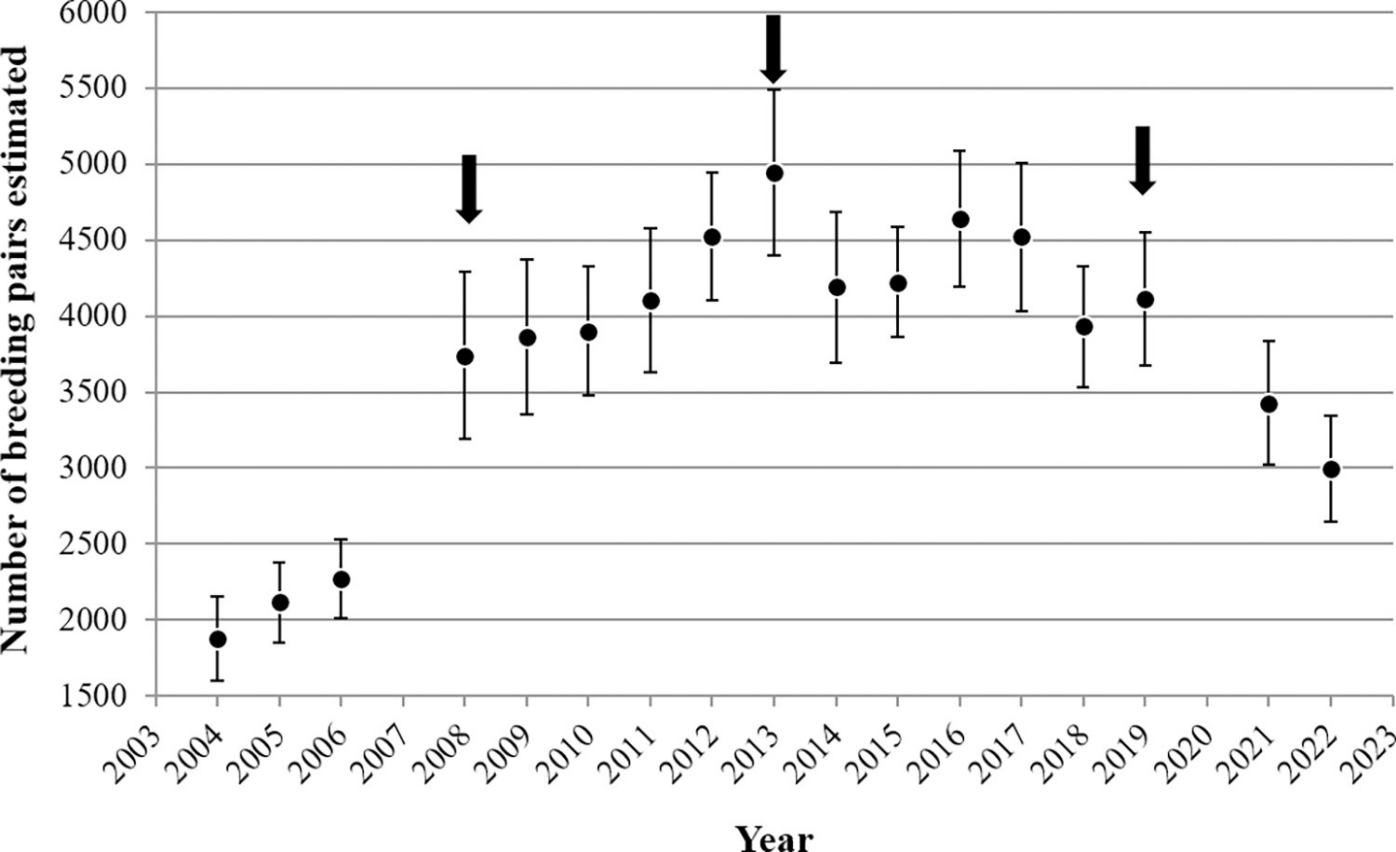
Colony size of the Magellanic penguin in Martillo Island. Total population (estimated number of breeding pairs for Distance Software) per year. Errors bars indicate ± SE. Arrows indicate the selected change points for model 2.

**Fig 4:**
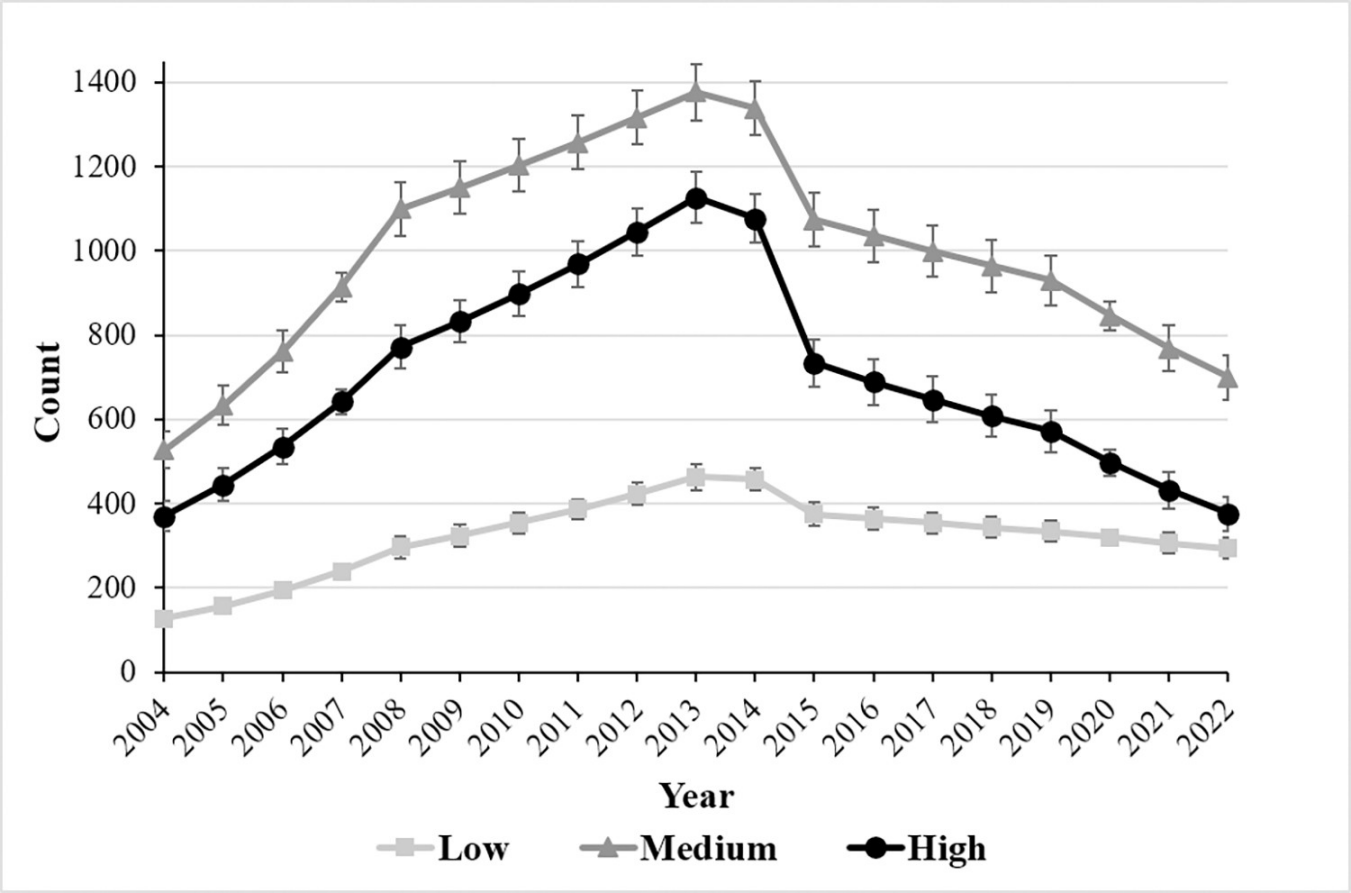
Growth trends of the Magellanic penguin colony on Martillo Island in each erosion zone and overall. Counted nest number per year and erosion zone: High, Medium and Low degrees of erosion. Mean and ± SE (errors bars) estimated on Model 2 using package rTRIM.

Between 2004 and 2022, overall colony size increased (Fig 3). The colony grew sharply until 2008 (69%). After reaching its peak of growth in 2013, it began to decline in 2014, coinciding with the westward expansion of the colony. Then remained stable, with fluctuations, but decreased over recent years (32% since 2019) (Fig 3). The first model (Model 1) estimated change points in each year, ignoring zone as a covariate (Table 1). This model showed significant changes in the slope (intensity of change) of the growth curves (β) in 2008, 2009, 2014 and 2019 years (Table 1), indicating different growth patterns throughout the study period. From 2004 to 2008, the increase was more rapid, then it grew more slowly until 2013, when the maximum colony size was reached. In 2014, there was a marked decrease, followed by years that oscillated between small increases and decreases in colony size, until after the last small increase in 2019, the colony began to decrease until 2022 (Table 1, Fig 3). The effect of zones on the change in abundance colony growth was evaluated in a more parsimonious model (model 2), selecting change-points at three years with a larger change in model 1 (2008, 2013, 2019, see Fig 3). Zone had a significant effect on changes in slope parameters (W_8_ = 20.85, p < 0.05), and these changes in slope were different for the three time periods (**2004–2008:** W_3_ = 96.17, p < 0.01; **2008–2013**: W_3_ = 23.38, p < 0.01; **2013–2019**: W_3_ = 41.64, p < 0.01; **2019–2022**: W_3_ = 5.99, *p* = 0.11; Fig 4).

**Table 1:** The growth rate (λ = multiplicative coefficients) and slope (β = additive coefficients) ± standard errors (SE) and 95% confidence interval (CI 95%). Wald test (W; with degree freedom = 1) for significance of changes in slope for the period between the years and its *p* value. * <0.05, ** <0.005, *** <0.001. Model 1 without zones as covariate). ^**a**^ Period of two years: 2006–2008 and 2019–2021.

Year		λ ± SE	CI 95%	β ± SE	CI 95%	W	*p*	
from	upto							
2004	2005	1.136 ± 0.081	[0.977, 1.295]	0.127 ± 0.071	[-0.012, 0.267]	3.20	0.074	
2005	2006	1.073 ± 0.072	[0.932, 1.214]	0.070 ± 0.067	[-0.061, 0.202]	0.26	0.611	
**2006**	**2008** ^**a**^	1.286 ± 0.046	[1.197, 1.375]	**0.252 ± 0.035**	**[0.182, 0.321]**	4.29	**0.038**	*
**2008**	**2009**	1.012 ± 0.054	[0.907, 1.118]	**0.012 ± 0.053**	**[-0.092, 0.117]**	10.57	**0.001**	**
2009	2010	1.070 ± 0.053	[0.966, 1.175]	0.068 ± 0.050	[-0.030, 0.166]	0.45	0.505	
2010	2011	1.016 ± 0.050	[0.918, 1.114]	0.016 ± 0.049	[-0.080, 0.112]	0.42	0.515	
2011	2012	1.114 ± 0.053	[1.010, 1.218]	0.108 ± 0.048	[0.015, 0.201]	1.36	0.244	
2012	2013	1.094 ± 0.050	[0.997, 1.192]	0.090 ± 0.045	[0.002, 0.179]	0.06	0.814	
**2013**	**2014**	0.845 ± 0.039	[0.769, 0.921]	**-0.168 ± 0.046**	**[-0.259, -0.078]**	12.53	**<0.001**	***
2014	2015	0.954 ± 0.046	[0.865, 1.043]	-0.047 ± 0.048	[-0.140, 0.047]	2.46	0.117	
2015	2016	1.058 ± 0.047	[0.965, 1.151]	0.056 ± 0.045	[-0.032, 0.144]	1.89	0.169	
2016	2017	0.973 ± 0.043	[0.888, 1.059]	-0.027 ± 0.045	[-0.114, 0.060]	1.33	0.250	
2017	2018	0.862 ± 0.040	[0.783, 0.941]	-0.148 ± 0.047	[-0.240, -0.056]	2.71	0.100	
**2018**	**2019** ^**a**^	1.012 ± 0.049	[0.916, 1.108]	**0.012 ± 0.048**	**[-0.083, 0.107]**	4.23	**0.040**	*
2019	2021	0.923 ± 0.027	[0.869, 0.976]	-0.080 ± 0.030	[-0.138, -0.022]	2.04	0.153	
2021	2022	0.856 ± 0.047	[0.764, 0.948]	-0.155 ± 0.055	[-0.263, -0.048]	1.10	0.293	

For the first growth period (2004–2008), the colony size increased in all zones. The increase was 19% in the high and medium erosion zones and 23% in the low erosion zone (Fig 4). During the second period (2008–2013), the growth was slower compared to the first period, reaching a maximum towards 2013 (between 4% and 12%). Like the previous period, the zone with the greatest increase was the low erosion zone (λ = 12%). The most stable was medium (λ = 4%) followed by high zone (λ = 8%). For the third period (2013–2019) colony size decreased slightly. The abundance in the colony decreased by 6% for the high zone, 4% for the medium and 3% for the low zones. For the last period (2019–2022), the estimated colony size decreased by 13, 9 and 4%, for high, medium and low zone, respectively (Fig 4). As observed, the effect of the zone is primarily attributed to the magnitude of the changes in slopes and growth coefficients, as the trends were similar across all three zones for each time period (Fig 4).

**Fig 5:**
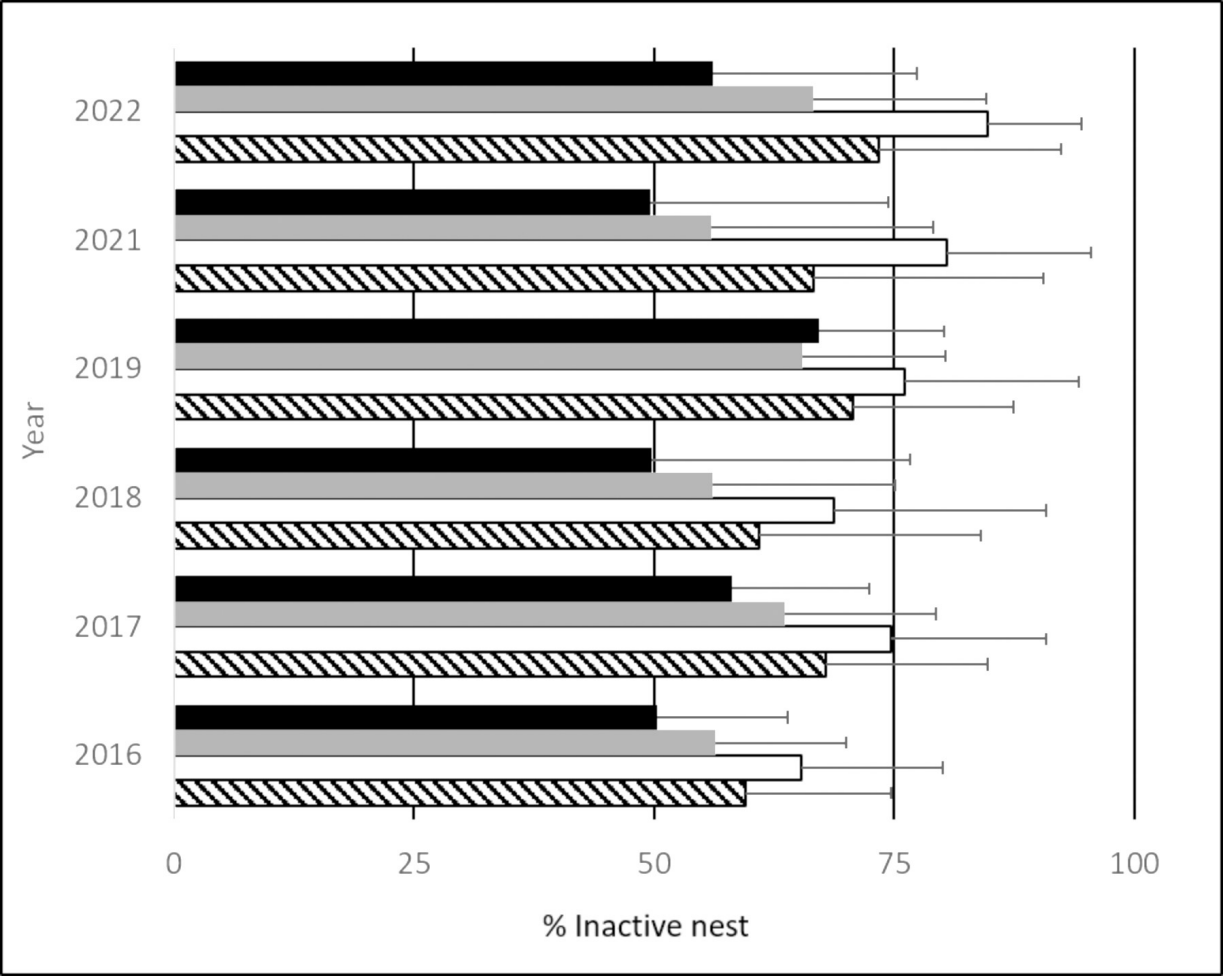
Percentage of inactive nests of Magellanic penguins per year in zones with high (black), medium (grey) and low (white) degrees of erosion and total (diagonal stripes). Mean ± SD (standard deviation = errors bars) are shown.

The percentage of empty nests was higher in the low erosion zone during all years compared to the high and the medium erosion zones (*H*_*2*_ = 114.87 *p* < 0.01; Low vs High z = 8.79, *p* < 0.01; Low vs. Medium z = 8.76, *p* < 0.01; Fig 5). On average over all years, 75% of the nests were empty in the low erosion zone, 61% in the medium erosion zone and 55% in the high erosion zone.

#### Body condition and health parameters

At the beginning of the breeding season (pre-laying), adult body condition differed among zones and did not differ between sexes within each zone (*F*_2_ = 10.29, *p* < 0.01; *F*_1_ = 0.16, *p* = 0.7, respectively). During this stage, adults from the medium erosion zone had a better body condition (Tuckey: *p* < 0.050, Table 2) than penguins from the low and high erosion zones (Table 2). In contrast, at the end of late chick-rearing breeding stage (CR), no differences in body condition were found between adults breeding in different zones (Table 2; *F*_2_ = 3.24, *p* = 0.06) and neither between males and females (*F*_1_ = 3.03, *p* = 0.10). When comparing the body condition throughout the breeding season within each zone, the body condition of adults from the high erosion zone at the CR was greater that at pre-laying (*F*_1_ = 9.91, *p* < 0.001; Table 2). On the contrary, the body condition of adults from the medium erosion zone decreased in CR with respect to pre-laying (*F*_1_ = 6.01, *p* < 0.05; Table 2), whereas in the low erosion zone the body condition of adults was similar at both stages (*F*_1_ = 0.65, *p* = 0.43; Table 2).

**Table 2:** Body condition and stress and breeding parameters of adult Magellanic penguins breeding in the three erosion zones (High, medium and low) at Martillo Island. Mean and SD (standard deviation) of body condition (adults body index), heterophyl vs. lymphocyte ratio (H/L), weight and body index of fledging, and breeding success are shown. Estimated coefficients (Est.), standard errors (se), t-values (with 21 degrees of freedom) and *p*-values (*p*) for specific comparisons of H/L between stages and between different degree zones, obtained from GLMM (to *F* see text) and z value from GLM with Poisson distribution order body condition over breeding success. ANOVA (*F*, with 2 freedom degree), Kruskal-Wallis (*H* with 2 freedom degree) statistics and their correspondent *p*-value. Significant differences are marked in bold. *N* number of birds (or nest for breeding success).

			**High**						**Medium**						**Low**							
**Body condition**								** **														
			**Mean (SD)**			**N**		** **	**Mean (SD)**				**N**	** **	**Mean (SD)**						**N**	
	**Pre laying**		-0.07 (0.26)			20			0.16 (0.32)				34		-0.21 (0.29)						20	
	**Chick rearing**		0.24 (0.47)			9			-0.09 (0.29)				13		-0.17 (0.26)						6	
			**High**						**Medium**						**Low**							
**H/L**			** **					** **							** **							
			**Mean (SD)**			**N **		** **	**Mean (SD)**				**N **	** **	**Mean (SD)**						**N **	
	**Pre-laying**		0.32 (0.11)			9			0.26 (0.09)				7		0.29 (0.05)						8	
											
	**Chick rearing**		0.46 (0.14)			9			0.30 (0.08)				7		0.34 (0.11)						8	
			**Est. (se)**	**t**_**21**_		***p***		** **	**Est. (se)**		**t**_**21**_		***p***	** **	**Est. (se)**			**t**_**21**_			***p***	
	**Pre-laying vs. Chick rearing**		0.17 (0.05)	3.13		0.01		*	0.09 (0.06)		1.37		0.18		0.05 (0.06)			0.89				
			**High vs. Medium**						**High vs. Low**						**Low vs. Medium**							
			**Est. (se)**	**t**_**21**_		***p***		** **	**Est. (se)**		**t**_**21**_		***p***	** **	**Est. (se)**			**t**_**21**_			***p***	
	**Pre-laying**		-0.1 (0.07)	-1.42		0.16			-0.02 (0.06)		-0.35		0.73		-0.07 (0.07)			-1.05			0.30	
	**Chick rearing**		-0.18 (0.07)	-2.71		0.01		*	-0.14 (0.06)		-2.21		0.04	*	-0.04 (0.07)			-0.57			0.58	
			**High**				**Medium**					**Low**										
			**Mean (SD)**			**N**	**Mean (SD)**			**N**		**Mean (SD)**				**N**	***F***_***2***_			***p***		
**Weight (Kg)**			3.6 (0.35)			42	3.75 (0.36)			82		3.6 (0.35)				38	2.24			0.11		
**Body index (bi)**			0.19 (0.02)			42	0.20 (0.02)			82		0.19 (0.02)				38	2.76			0.07		
** **																	***H***_***2***_			***p***		
**Breeding success Ch/N**			1.33 (0.89)			61	1.18 (0.9)			174		1.38 (0.75)				39	1.67			0.3		
		Body condition (pre-laying)	**Est. (se)**			N											***z***			***p***		
			- 0.18 (0.38)			61											0.49			0.63		

Heterophyl vs. lymphocyte ratio (H/L), linked to stress levels, was different between stage and erosion zone (*F*_1,21_ = 10.09, *p* < 0.01 and *F*_1,21_ = 3.59 *p* < 0.05, respectively). H/L was higher during chick rearing than before egg laying for individuals from high erosion zone (Table 2). During chick rearing, H/L was higher for individuals at the high erosion zone than the other two erosion zones (Table 2). While during pre-laying, H/L was similar among individuals from the three zones (Table 2). Identity accounted for 35% of the variability.

### Foraging behavior and trophic parameters

The foraging effort of penguins from the high erosion zone was higher for some parameters. Particularly, the dive rate was higher for penguins from the high compared with the medium zone, and the percentage of time diving was greater in the high compared with the medium and low zones (Tukey test, *p* < 0.05 for both parameters). However, time spent at the bottom per trip, trip duration and maximum distance were similar among individuals across the different zones either (Table 3). The sinuosity of the foraging path did not differ among zones (Table 3). Most of the individual feeding areas were located to the east of Martillo Island, except for one penguin from the low erosion zone that foraged to the west (maps in S2 Fig).

**Table 3:** Foraging effort, feeding efficiency and movement parameters of Magellanic penguins in the three erosion zones (High, medium and low) at Martillo Island. Mean ± SD (standard deviation) are shown. ANOVA statistics (*F*, with 2 degrees of freedom) and their correspondent *p*-value for differences in each parameter between zones are shown. Significant differences are marked in bold. *N* number of birds. ^a^ N = 6 and ^b^ N = 7 for Maximum distance and Sinuosity.

	High		Medium		Low			
	N = 5^a^		N = 6^b^		N = 5			
	Mean ± SD	Range	Mean ± SD	Range	Mean ± SD	Range	*F*_2_	*p*
Trip duration (h)	30.6 ± 11.4	17.2–48.3	21.3 ± 4.7	13–27.1	22.8 ± 8.3	13.3–32.3	1.86	0.194
Dive rate (dives/h)	52.9 ± 9.8	37.8–63.1	37.1 ± 3.7	30.7–40.6	39.6 ± 8.8	29.6–49.9	6.46	**0.011**
Bottom time (min/h)	6.7 ± 1.4	4.8–8.5	5.1 ± 1.1	3.3–6.7	5.5 ± 1.9	4.1–8.7	1.67	0.226
Time diving (%)	51 ± 8.4	38.5–60.9	40.7 ± 4.6	36.7–48.1	43.1 ± 4.9	38.0–50.3	4.14	**0.041**
Maximum distance (km)	54.9 ± 17.1	32.1–75.8	42.6 ± 18.3	15.9–60.1	40.5 ± 11.7	18.1–52.2	1.52	0.250
Sinuosity	1.2 ± 0.13	1.1–1.4	1.2 ± 0.2	1.1–1.4	1.2 ± 0.1	1.1–1.5	0.06	0.942

**Fig 6:**
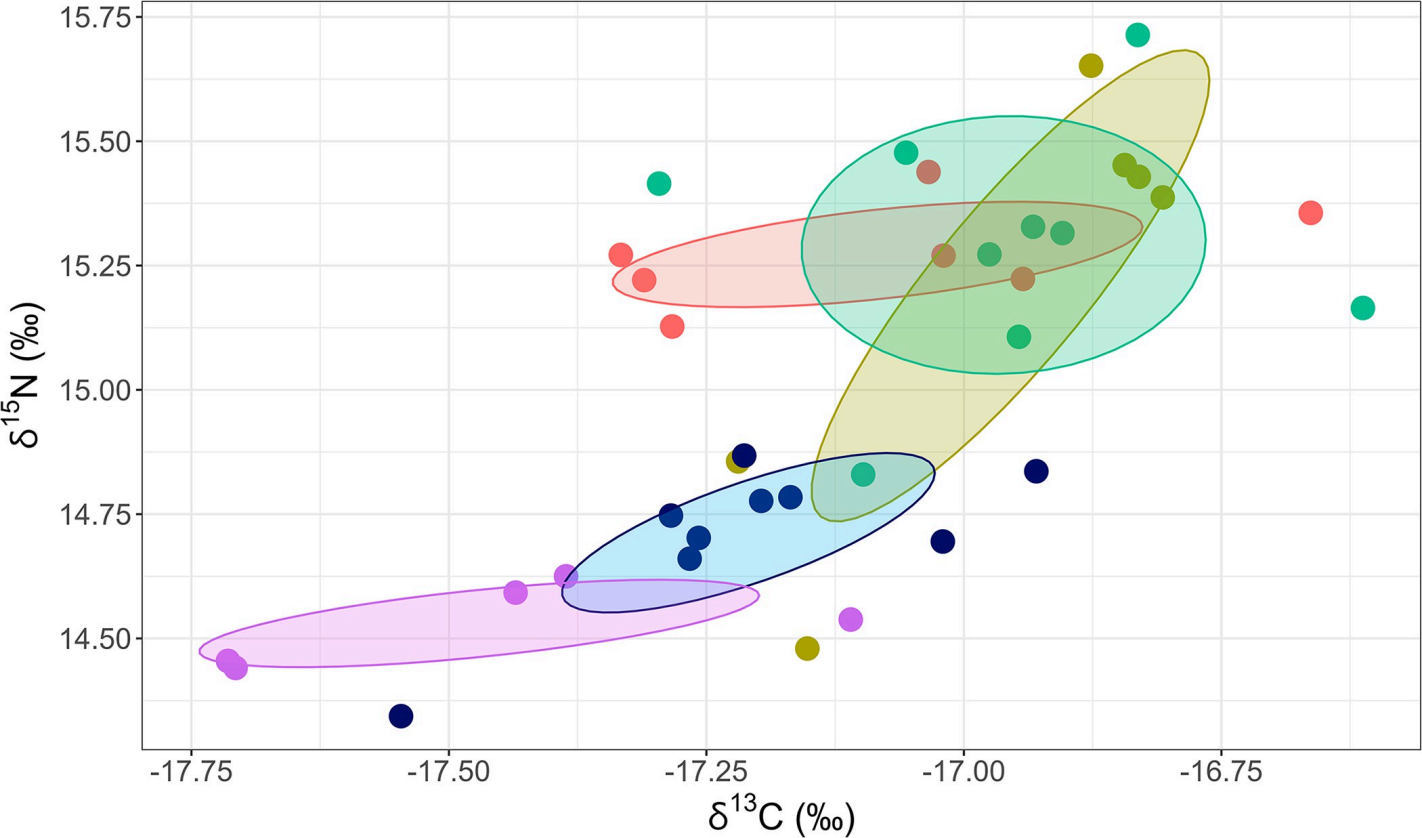
Stable isotope signatures of Magellanic penguins in 2017 at Martillo Island. Carbon and nitrogen stable isotope biplot and standard ellipse areas corrected for small sample size (SEA_C_) of blood adult *n* = 22 from zones with different erosion degrees: high (red), medium (green) and low (yellow) and fledging chicks *n* = 14 from high (blue) and medium (purple) erosion zone.

Stable isotope signatures in blood of adult Magellanic penguins showed no difference in mean δ^13^C and δ^15^N values (representing foraging area and diet, respectively) among zones (*F*_2_ = 0.88, *p* = 0.43; *H*_2_ = 0.15, *p* = 0.77, respectively, Fig 6). However, mean δ^13^C and δ^15^N values for fledging chick blood differed among zones (*F*_1_ = 4.92, *p* < 0.05; *F*_1_ = 5.3, *p* < 0.05, respectively). Fledging chicks from the high zone presented slightly higher δ^13^C and δ^15^N values (-17.20 ‰, SD = 0.07; 14.71 ‰, SD = 0.05, respectively) than medium zone chicks (-17.47 ‰, SD = 0.09, 14.53 ‰, SD = 0.06, respectively). In addition, mean δ^13^C and δ^15^N values differed between adults and fledging chicks in the medium zone (*F*_1_ = 18.64, *p* < 0.01; *F*_1_ = 1.86, *p* < 0.01, respectively), registering higher isotopic values in adults than in chicks for both δ^13^C and δ^15^N (mean δ^13^C: -16.96 ‰, SD = 0.07 vs. -17.47 ‰, SD = 0.09 and mean δ^15^N: 15.29 ‰, SD = 0.07 vs. 14.53 ‰, SD = 0.09, respectively). In the high erosion zone, we only found differences between adults and chicks in the mean δ^15^N values (*F*_1_ = 60.69, *p* < 0.01), being higher in adults (15.27‰, SD = 0.05) than in chicks (14.81‰, SD = 0.05), but not in mean δ^13^C values (*F*_1_ = 1.16, *p* = 0.3). In adult Magellanic penguins, the variances of nitrogen isotopic signatures in blood were not homogeneous among the different zones, being more variable for the low zone compared to the others (High vs. Low: *F*_1_ = 0.05, *p* < 0.01; High vs. Medium: *F*_1_ = 0.16, *p* < 0.05).

### Breeding parameters

**Fig 7:**
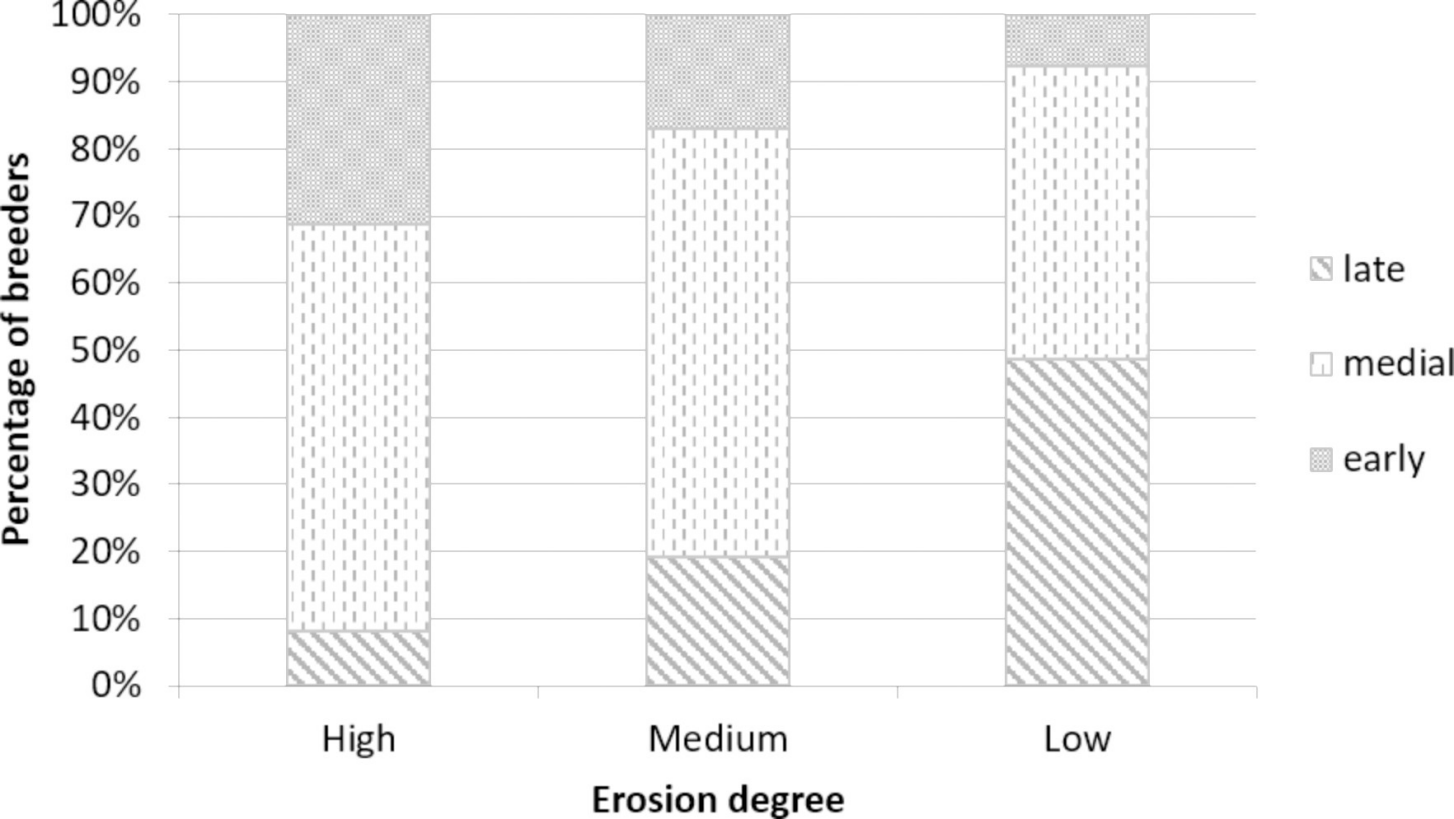
Percentage of early, medial or late breeders (nests) for each zone with high, medium or low erosion degrees.

The low erosion zone had the highest proportion of late breeders. The highest proportion of early breeders was found in the high erosion zone, followed by the medium erosion zone (Fig 7, *X*^*2*^_4_ = 26.37, *p* < 0.01, n = 277).

No differences were found in fledging weight (w) and body index (bi) among zones (Table 2). Thus, for the entire colony, the weight and body index of fledging chicks were 3.7 kg (SD = 0.36, n = 162) and 0.19 (SD = 0.02, n = 162). Mean breeding success for the Magellanic penguin colony was 1.24 Ch/N (SD = 0.88, n = 274). Success was not affected by the body condition of individuals in pre-laying and no differences were detected amongst zones (Table 2).

## Discussion

Magellanic penguins have been nesting on Martillo Island since at least 1976 and there is a possibility that they originate from a colony present until the 1950´s about 60 km away from Martillo Island in the Beagle Channel [58]. During the study period, nest density remained higher in the northern and south-eastern sectors of the island, and the colony gradually expanded its distribution westwards. Changes in nest abundance varied between nesting zones with different occupation time, density, and erosion. The decline observed in the densest, most eroded zone and with earliest occupation suggests that environmental degradation may impose a limit on the carrying capacity of the colony. The recent expansion of the colony to previously unoccupied areas suggests a dynamic within the colony that may allow the colony persistence. The most eroded areas, where density is reduced, can recover at least partially, as the vegetation can slowly re-establish while the substrate would remain unstable. Compare the trophic and reproductive characteristics, and the body condition and stress of individuals among zones allows us to understand if the observed expansion is carried out by new breeders or by more experienced individuals moving from eroded areas.

### Colony trends

Magellanic penguins have experienced population fluctuations and an expansion of their distribution in the last years [7, 59–62]. At Martillo Island, the initial establishment and persistence of nesting in the high density area could be related to the shelter from the prevailing south-westerly winds provided by the hill and the forest surrounding this sector, rather than to the characteristics of the nesting substrate (see detail of degrees of erosion and characteristic of nesting areas in [42]).

As noted above, density trends over the years differed among zones of Martillo island, particularly in the latter years, when density declined more steeply after a short period of relative stability within the high and medium erosion zones, being more evident in the high erosion zone. Population regulation in seabirds involves both density dependent and independent factors [63]. Density dependence in population growth has been described for Magellanic colonies in northern Patagonia, where smaller and more recently established colonies present higher growth rates, which has been attributed to a shift in the anchovy distribution [7]. In this study, a density-dependent effect may be occurring on Martillo Island, due to degradation of nesting habitat. Bio-erosion reduces the quality of nesting areas, at least in terms of available free space [42] for dig new burrows. In the low erosion zone, the most recently colonized sector of the island and with lowest nest density, the growth was higher during periods of increase but was lower (close to zero) during periods of decline, compared with the other zones. The different growth patterns among zones may arise as individuals from the denser and more eroded areas move to other zones in the following breeding seasons in search of better nesting sites. Moving to new breeding sites may be driven by competition for nesting sites, when habitat quality (for example, nest-site quality) decreases at established sites (The "Habitat Quality" hypothesis: [7, 64] or if poor or less experienced competitors increase their fitness by pioneering new sites (“Individual Quality” hypothesis: [7, 64]. According to the “Habitat Quality” hypothesis, if newer sites are colonized because of a decrease in habitat quality at older sites, this would be reflected in lower productivity, higher nest density and declining population trends at the old sites. On the other hand, the “Individual Quality” hypothesis predicts that, given the experience of breeders productivity at newer sites would be lower than at older sites, despite the potential fitness improvement for individuals that relocate, because they are of lower quality compared to those that remain in older areas and the colony trends would increase at older and denser sites [64].

This study reveals that the greatest decrease in density was observed in the most eroded and densely populated zone, which is consistent with predictions of the habitat quality hypothesis. However, the reproductive success was not significantly lower in the most eroded areas, as predicted by the individual quality hypothesis. Both hypotheses may have a combined effect, is either cumulative or interdependent, which means that they are not mutually exclusive. While we can assess which hypothesis aligns better with our findings, we can´t reject entirely either hypothesis [64]. Therefore, we cannot reject the habitat quality hypothesis, as similarities in reproductive success may be attributed to the lack of limitations in resources that drive reproductive success, such as food abundance and availability.

Dispersal of individuals, which plays an important role in population dynamics, is a flexible trait that varies in time and space depending on environmental and individual conditions [3, 65, 66]. Magellanic penguins exhibit high nesting area and site fidelity, which would provide little support for the “Habitat Quality” predictions [67]. At Martillo Island, adults that were marked and recaptured the following season showed high nesting site fidelity [68]. This was only studied in one season and in reproductively successful individuals, so the possibility should not be ruled out that under greater resource limitation, site fidelity becomes less valuable and individuals, whether new or old breeders, move away from nesting sites.

Recruitment and movements of younger breeders to non-natal colonies or areas has been recorded in several seabird species [69–71], despite the fact that new breeders are known to be faithful to their natal site [7, 18, 64, 72, 73]. It has been suggested that recruitment of juvenile Magellanic penguins to the newest northern colonies from Patagonia involves animals dispersing from southern colonies. New colonization involves a trade-off between higher quality breeding habitat than their previous breeding site and increased predation risk due to the loss of the "dilution effect" of breeding in large numbers [74].

At present, we have no evidence that tagged breeders from the most eroded areas migrate to less eroded areas in subsequent seasons, and we have incomplete information on whether tagged chicks breed near their natal site or in other zones of the island, as these data are currently being analysed. However, cases of penguins nesting away from their native habitat have been documented (Scioscia et al. data not pub.). In addition, a comparative analysis of reproductive and trophic characteristics of breeding individuals may provide insight into whether they are new or experienced breeders, as discussed in more detail below.

### Body condition, stress, trophic and foraging behavior and breeding by erosion zone

New and less experienced breeders typically exhibit differences in reproductive and feeding performance compared to older and more experienced individuals [40, 75, 76]. In addition, in situations of limited resources, younger individuals may prioritize self-maintenance over breeding. Therefore, if their body condition is compromised, they may abandon a breeding event to preserve their future breeding potential [77]. As individuals get older, they become more efficient in their foraging, may begin breeding earlier and become more proficient in their parental duties, thereby reducing the effort of the breeding event [70, 78]. Furthermore, as individuals age and the likelihood of future breeding events diminishes, breeding would be favored over self-maintenance and consequently may allocate more resources towards chick rearing even at the expense of their own body condition [79].

Body condition can be measured by indexes of size versus weight [50, 80] and also by their immunological state such as long-term stress level reflected in the heterophil vs. lymphocyte counts [52, 81–84]. Therefore, individuals with equivalent breeding outputs for a given breeding season may have endured different costs reflected in their body condition or immunological state which could affect their performance during migration and ultimately, carry-over to future breeding events [85].

In seasons with high abundance of prey, adults can provision their chicks with a low impact on their own condition. If prey availability is reduced, adults may incur higher foraging costs (longer trips, higher effort), internal costs such as increased stress or poorer body condition, or even breeding costs such as producing lighter fledglings or reduced breeding success [86, 87]. Younger individuals tend to be less proficient in their foraging skills [38, 41, 88] which may influence whether they to initiate or persist in the breeding season. During reproduction, adults can either provide energy-rich prey to their growing offspring, thereby incurring in higher costs, or they can consume lower quality prey that is more accessible, therefore minimizing reproductive costs even at the expense of the growth and survival of their offspring [39, 89, 90].

On Martillo Island, younger and new breeders would be prone to settle in a new area on the island, occupying vacant spaces towards the west. This is supported by a higher percentage of later breeders occupying the less eroded and more recently inhabited area, considering that in other seabird species laying date seems to be linked to age of breeders [70, 91]. In turn, the higher number of empty nests found in the new area may be due to increased abandonment by inexperienced adults, either due to difficulty finding a mate or to a failed breeding attempt. Observations show that individuals initially occupy nests without mates or eggs, but by the peak of egg-laying, they have vacated the nests or formed pairs but subsequently abandoning the eggs (Scioscia, pers. Obs.). This reproductive failure is more common in younger or inexperienced individuals [70, 75, 92, 93]. The higher number of empty nests in the new area suggests that this site may be more likely to be occupied by young breeders.

The higher proportion of early and medial breeders in the high erosion zone suggests that they are older or more experienced individuals. However, the medium erosion zone, which is the most represented in the colony, has a greater number of medial breeders, with a smaller but similar proportion of late and early breeders, suggesting a mixture of older (sum medial and early breeders) and fewer younger breeders (late breeders).

On the other hand, we suggest that the medium and high erosion zones may hold either more older breeders that endure higher costs to breed successfully, or individuals facing higher reproductive costs as direct or indirect consequences of bio-erosion. This effect of habitat degradation on nesting sites may have a direct impact on chick survival, such as increased susceptibility to flooding or landslides. In addition, it may indirectly create unfavorable habitat conditions, such as increased parasite load, which could affect the body condition and health of adults and reduce their ability to effectively feed their chicks.

The higher cost of chick rearing, evidenced by changes in body condition, was more pronounced in the medium erosion zone. The individuals had the highest decrease in body condition throughout the season, generating a higher cost of reproduction (marginally evidenced in lower breeding success). These individuals exemplify how reproduction can take a toll on body condition without making a detectable difference in breeding outcome, stress or foraging effort. Future studies will try to determine if each erosion zone has differences in the proportions of each age group and if differences in foraging, body condition and breeding parameters may be age-related. On the other hand, for the high erosion zone, the cost of rearing chicks added to the cost of improving the adult body condition, and was reflected in an increase in stress and foraging effort at the end of the season. These individuals from high erosion zone invested more in their foraging behavior to obtain isotopically equivalent diets, as isotopic signals did not differ between zones.

Chicks from the high erosion zone had slightly higher δ^13^C and δ^15^N isotopic values than chicks from the medium erosion zone. The Magellanic penguins from Martillo island feed mainly on Fuegian sprat, *Sprattus fuegensis*, Squat lobster *Grimothea gregaria* and Patagonian squid *Loligo gahi* [94]. Fuegian sprat has higher δ^13^C and δ^15^N values than pelagic Squat lobster [95]. Therefore, chicks from the high erosion zone may have been fed “higher quality” sprat at the cost of higher foraging effort of adults. Isotopic signatures encompass the diet within -at least—the previous month, which may have merely compensated for any previous deficiencies in chick growth, allowing them to reach a similar weight at fledging and reproductive success as those from other zones. This compensation has been observed in this colony during "poor" years [50].

Younger and less experienced birds may face higher breeding costs than more experienced breeders, partly due to foraging skills and parental duties [96]. The lack of evidence in our study supporting this would be because only successful breeders were studied. Less experienced individuals may abandon the breeding attempt at early stages before enduring any investment in chick rearing [93]. Other reasons could be that reproductive success did not vary between zones because other resources, such as food availability, are not limiting in that region, or that there are not terrestrial predators in the colony.

Future research will aim to include failed breeders from each zone to determine if any aspects of body condition or stress and foraging behavior may be triggering the abandonment of the breeding attempt.

Bio-erosion on Martillo Island is produced by guano, trampling, removal of vegetation by the penguins and by other species such as the introduced muskrat. Erosion also depends on terrain characteristics such as soil, slope and geographical orientation, which will determine how many penguins can occupy each sector of the Island [42]. In addition to the risk of burrow collapse, erosion may reduce vegetation cover which in turn may make substrate dryer and warmer and hence more suitable for endo- and ectoparasite infestation [45, 97, 98]. Furthermore, a higher nest density may also favour the transmission of parasites [99]. Therefore, a combination of soil characteristics and nest density might contribute to nest abandonment in more eroded areas or render a particular zone less suitable, increasing the costs of reproduction for resident penguins and forcing recruits to find new nesting sites within the colony.

### Concluding remarks

Life history theory predicts reproduction occurs when benefits outweigh costs in the trade-off between fitness and reproduction throughout their life [12]. In this study, except for pre-laying body condition, we only considered individuals that were reproducing and, therefore, with good enough overall condition to start a breeding event during the studied season. Once individuals form a nest and begin breeding, they maintain high site fidelity. Therefore, the observed reduction in breeding pairs in the “older” sections of the island could mainly represent individuals skipping a breeding event or even reaching senescence and/or dying off. New individuals recruiting into the breeding colony could be choosing less eroded sections of the island, either to reduce competition for nests, or to avoid other effects of erosion and of high density (flooding, ectoparasite prevalence, etc). If this is the mechanism behind the shifting in numbers throughout the island, we expect the island will progressively become more occupied to the west, and unoccupied to the east. If competition or other density dependent factors are in play, a time will come when the vacant east side will slowly be recolonized by younger individuals. If erosion or other longer time effects are at play, recolonization may not occur in the foreseeable future and the colony may ultimately be abandoned as individuals search for new breeding grounds. Therefore, erosion at the breeding site may be a key factor in population trends of this burrow-nesting species. Magellanic penguins are known to colonize new sites throughout their distribution [7, 67], yet the dynamic within a given colony are poorly understood. Martillo Island is today the only Magellanic penguins breeding ground at the Beagle Channel. In addition, there is a large colony on Observatory Island, and three smaller in Isla de los Estados and Año Nuevo Islands, Argentina, and in the Murray Channel, Chile. It would be interesting to evaluate whether there is a flow of individuals between these colonies and, on the other hand, to carry out a study of suitable habitats to determine whether there are other uninhabited sites with the necessary characteristics for an eventual new colonization in nearby sites. The apparent origin of the Martillo island penguins from a colony located in the Beagle Channel 50 years ago, suggests that the species presents a spatial dynamic where local colonization and extinction are relevant process that would ensure the persistence of the regional population. This work reveals the role of the erosion in this spatial dynamic process.

Further research is crucial to understand specific mechanisms driving colony dynamics to inform targeted conservation actions. Managing degraded habitats should involve mitigating factors exacerbating nest collapse, such as regulating tourist visits or constructing elevated walkways. Additional measures may include erosion mitigation by eradication of introduced muskrats.

## Supporting information

S1 Fig Percentage of nests with 1 egg according to its laying date (bars) and percentage accumulated (line). (TIF)

S2 Fig Map of the feeding areas of Magellanic penguin nesting at zones with HIGH, MEDIUM and LOW degrees of erosion at Martillo Island. The contour shapefiles of Tierra del Fuego (Argentina: dark grey and Chile: light grey) were obtained from the National Geographic Institute of the Argentine Republic (IGN), https://www.ign.gob.ar.
(TIF)
