# Supplementary figures and images for: Do penguins care about their neighborhood? Population implications of bioerosion in Magellanic penguin, Spheniscus magellanicus, at Martillo Island, Beagle Channel, Argentina
Source: PLoS One. 2024 Nov 19;19(11):e0310052. doi: 10.1371/journal.pone.0310052 (PMC11575772; doi:10.1371/journal.pone.0310052)

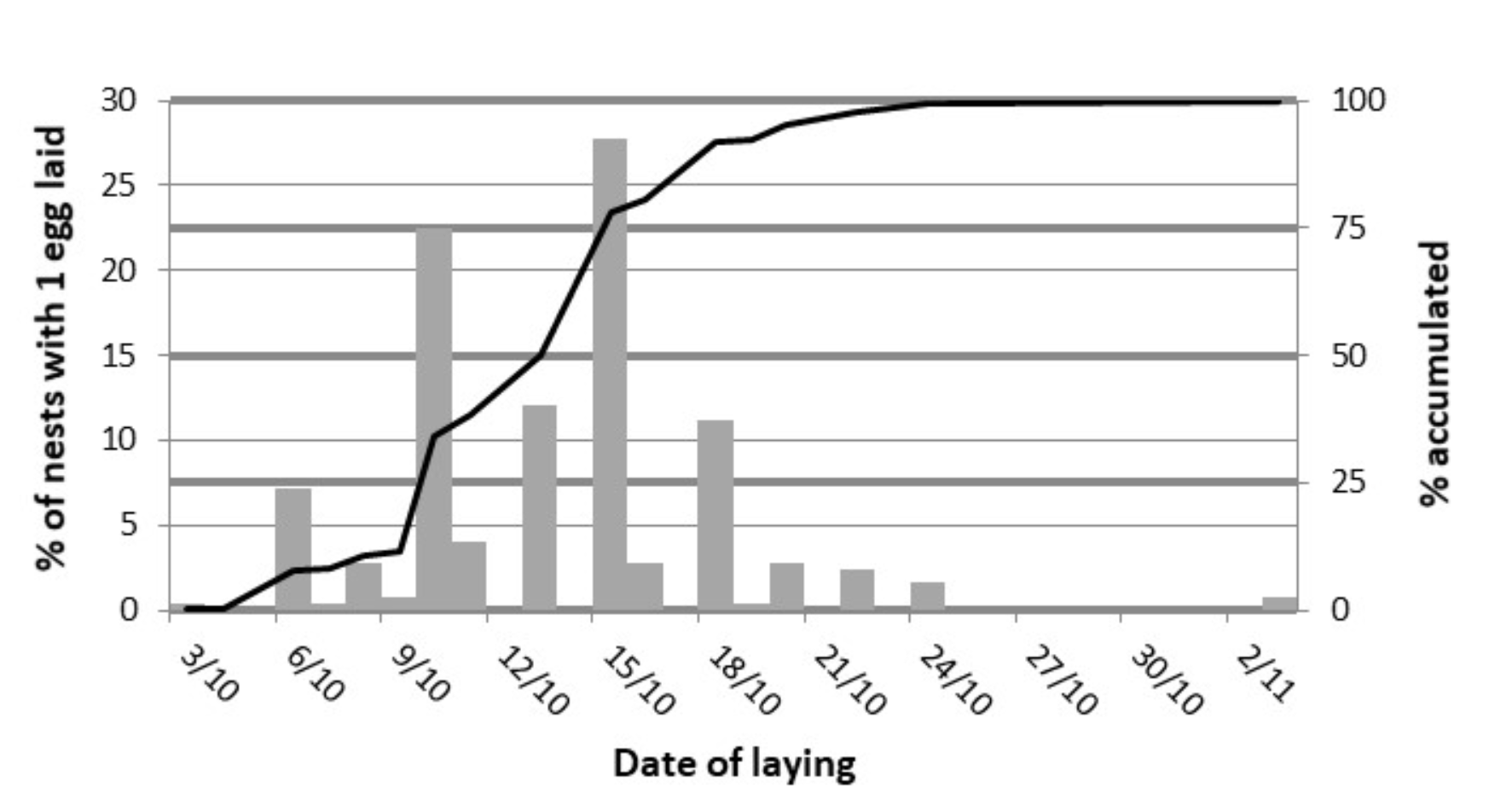

Supplement: S1 Fig — (TIF) [file pone.0310052.s001.tif]

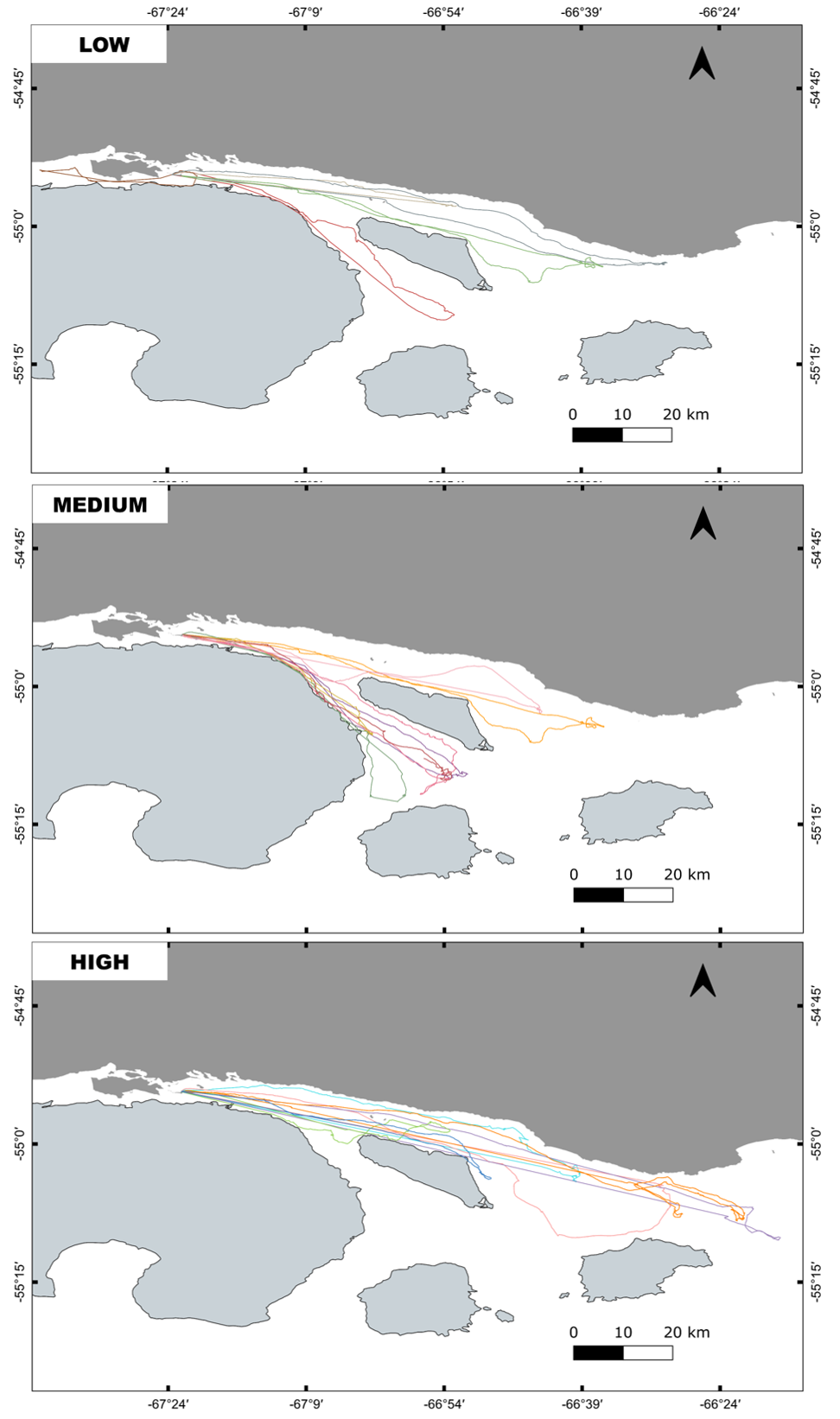

Supplement: S2 Fig — The contour shapefiles of Tierra del Fuego (Argentina: dark grey and Chile: light grey) were obtained from the National Geographic Institute of the Argentine Republic (IGN), https://www.ign.gob.ar. (TIF) [file pone.0310052.s002.tif]
